# Quantitative MRI Assessment of Orbital Structures in Radioactive Iodine‐131 Therapy for Inactive Graves’ Ophthalmopathy

**DOI:** 10.1155/ije/8200828

**Published:** 2026-04-26

**Authors:** Man Wang, Zhong-Yuan Shen, Xing-Wang Wu

**Affiliations:** ^1^ Radiology Department, The First Affiliated Hospital of Anhui Medical University, Hefei, Anhui Province, China, ahmu.edu.cn; ^2^ Nuclear Medicine, Fuyang Hospital Affiliated to Anhui Medical University, Fuyang, Anhui Province, China; ^3^ Radiology Department, Fuyang Hospital Affiliated to Anhui Medical University, Fuyang, Anhui Province, China

**Keywords:** diffusion-weighted imaging, Graves’ ophthalmopathy, magnetic resonance imaging, single-photon emission computed tomography/computed tomography

## Abstract

**Objective:**

The understanding regarding the role of orbital magnetic resonance imaging (MRI) in predicting the prognosis of radioactive iodine‐131 (I‐131) therapy for patients with inactive Graves’ ophthalmopathy (GO) is limited. Therefore, this study aimed to explore the application of MRI quantitative measurements of orbital structures in radioactive I‐131 therapy for inactive GO.

**Methods:**

This retrospective study included 41 patients (82 eyes) diagnosed with GO at Anhui Medical University Affiliated Fuyang Hospital and Anhui Medical University First Affiliated Hospital from January 2022 to October 2025 as the GO group, and 32 patients (64 eyes) with Graves’ disease (GD) who were matched with the GO group in terms of gender and age were selected as the GD group. All patients were treated with radioactive I‐131 using the calculated dose method. MRI conventional images and readout segmentation of long variable echo‐trains diffusion‐weighted imaging (RESOLVE DWI) were performed before and after I‐131 treatment, as well as the thyroid technetium (^99m^TcO4‐) imaging using single‐photon emission computed tomography/computed tomography (SPECT/CT), and a correlation analysis was conducted between the changes in orbital MRI parameters and thyroid weight in GO patients. All eyeball parameters are taken as the average of the two eyes.

**Results:**

Before radioactive I‐131 treatment, there were statistically significant differences in the average values of apparent diffusion coefficient (ADC) of the lacrimal gland and the long diameter of the transverse lacrimal gland between the GO group and the GD group (*p* ≤ 0.01). However, there was no statistically significant difference in the coronal lacrimal gland parameters of MRI and the thyroid parameters of SPECT/CT (*p* > 0.01). There was a statistically significant difference in the MRI orbital parameters (lacrimal gland protrusion [13.65 ± 1.47 vs. 12.02 ± 1.25], the long diameter [17.21 ± 2.29 vs. 16.03 ± 228], and area [93.05 ± 15.66 vs. 84.68 ± 14.76] of the lacrimal gland in the cross‐section; longitudinal diameter [17.99 ± 2.71 vs. 17.67 ± 2.61] and area [109.81 ± 20.52 vs. 104.50 ± 19.03] of the lacrimal gland in the coronal plane) between the GO group before and after radioactive I‐131 treatment (all *p* < 0.001). In addition, thyroid weight (53.53 ± 27.28 vs. 48.22 ± 23.65, *p* < 0.001), thyroid area (28.08 ± 10.25 vs. 24.89 ± 8.74, *p* < 0.001), and thyroid technetium uptake (17.82 ± 11.34 vs. 15.14 ± 9.57, *p* < 0.001) measured by SPECT/CT were also decreased. The change values of lacrimal gland protrusion (*r* = 0.600), the mean ADC (*r* = 0.766), the long diameter (*r* = 0.748), and area (*r* = 0.678) of the transverse lacrimal gland and the long diameter (*r* = 0.683) and area (*r* = 0.560) of the coronal lacrimal gland in the GO group before and after radioactive I‐131 treatment were positively correlated with the change values of thyroid weight (all *p* < 0.05).

**Conclusion:**

In patients with GO, lacrimal gland protrusion, MRI lacrimal gland length and area, and mean lacrimal gland ADC value may be reliable imaging indicators for evaluating the I‐131 treatment.

## 1. Introduction

Graves’ ophthalmopathy (GO), also known as thyroid eye disease or thyroid‐associated ophthalmopathy, is the primary extrathyroidal manifestation of Graves’ disease (GD) [1]. The incidence of GO ranks first among autoimmune eye diseases [2]. It is usually associated with hyperthyroidism and rarely with hypothyroidism or normal thyroid function [3]. GO is more commonly seen in women [4], while male patients tend to have more severe symptoms [5]. Most patients with GO have mild conditions that are relatively stable or spontaneously regress [6]. Due to the lack of specific symptoms, GO is often underdiagnosed or misdiagnosed. The average duration of GO and thyroid dysfunction in Chinese patients is significantly longer than in European patients [7], but only 5%–6% of cases are moderate to severe [8]. The exact causes and mechanisms of GO remain unclear, making its management challenging [9]. GO management in clinical settings currently depends on assessing the inflammatory activity and severity [10, 11]. Patients with GO in the active phase are not suitable for radioactive iodine‐131 (I‐131) treatment [12]. Radioactive I‐131 treatment should be considered only after the hormonal levels of hyperthyroidism have been well controlled, symptomatic treatment for the eyes has been carried out, and the patients have reached the inactive phase [12]. Therefore, early diagnosis is crucial for GO.

The lacrimal gland, like the extraocular muscles, appears to be an important target in the autoimmune reaction process of GO [13–15]. Gao et al. [16] conducted orbital computed tomography (CT) scans on patients with GO and observed an enlargement of the lacrimal glands. Magnetic resonance imaging (MRI) was also used to measure the lacrimal gland structure in patients with GO, showing that MRI can assist in diagnosing and staging GO [17]. Deng et al. [18] studied the changes and significance of thyroid volume, technetium uptake, and thyroid function in patients with GD after I‐131 treatment. Moura‐Neto et al. [19] conducted a study on the predictors of failure of I‐131 treatment for GD. They reported that functional thyroid quality was the most definitive factor in treatment failure.

Although various methods and approaches exist to evaluate GO, there is still a lack of standardized measurements and uniformity in analyzing certain treatment outcomes. The extent of benefit for patients with inactive GO undergoing I‐131 treatment is not well quantified. In addition, the value of orbital MRI in assessing the prognosis of I‐131 treatment in patients with inactive GO, particularly concerning the lacrimal gland, remains poorly understood. Therefore, this study aimed to explore the application of MRI quantitative measurements of orbital structures in radioactive I‐131 therapy for inactive GO.

## 2. Materials and Methods

### 2.1. Study Design and Participants

This retrospective study included patients diagnosed with GO at Fuyang Hospital Affiliated to Anhui Medical University and the First Affiliated Hospital of Anhui Medical University from January 2022 to October 2025. The inclusion criteria were as follows: (1) patients who had not yet received hormone shock therapy, immunosuppressive therapy, radiotherapy, or surgical treatment, (2) during the hospitalization of GO patients for I‐131 treatment and during the 1‐year follow‐up period when thyroid function was normal or hypothyroidism was detected, MRI + readout segmentation of long variable echo‐trains diffusion‐weighted imaging (RESOLVE DWI) and thyroid technetium (^99m^TcO4‐) imaging scans were both performed with image quality sufficient for further analysis, and (3) patients who were clinically diagnosed with inactive disease. The exclusion criteria were patients (1) with other eye diseases such as retrobulbar space‐occupying lesions, ocular trauma, hypertension, diabetes, glaucoma, cataracts, lacrimal gland‐related diseases, etc., (2) who previously received orbital radiotherapy or orbital decompression surgery, or (3) with other severe conditions (heart failure, respiratory failure, malignant tumors, etc.). This study complied with the Declaration of Helsinki and was approved by the Ethics Committee of Fuyang Hospital Affiliated with Anhui Medical University (approval #KY2024068). The requirement for individual consent was waived by the committee because of the retrospective nature of the study.

The diagnosis of GO was based on the Bartley diagnostic criteria [20]: (1) presence of eyelid retraction, (2) abnormal thyroid function, characterized by elevated serum levels of free T3 and free T4, and decreased TSH levels, (3) proptosis (eye bulging) of no less than 20 mm, (4) optic nerve dysfunction that cannot be explained by other lesions, and (5) involvement of extraocular muscles resulting in restricted eye movement, confirmed by imaging to show enlarged extraocular muscles. For individuals with eyelid retraction, at least one among #2, #3, #4, and #5 had to be met as part of the inclusion criteria. In the absence of eyelid retraction, a diagnosis of GO could be made if criterion #2 was met, along with at least one among #3, #4, and #5. The inactive disease was evaluated by clinical activity scores (CASs). Clinical activity was assessed using the modified 7‐point CAS [21]. The evaluation was performed on a per‐eye basis. A CAS score of ≥ 3 indicated an active disease. A CAS score of < 3 indicated an inactive disease.

### 2.2. Data Collection and Definition

In this study, all patients with GO and GD treated with radioactive I‐131 were hospitalized. The GO and GD data are derived from medical records and included age (year), gender, time of orbital MR examination and reexamination time (day), and the CASs of both eyes of the patients. Before I‐131 treatment, all patients completed five thyroid function tests (thyroid‐stimulating hormone [TSH], triiodothyronine [TT3], thyroxine [TT4], free triiodothyronine [FT3], and free thyroxine [FT4]) and thyroid autoantibody tests (thyrotropin receptor stimulating antibody [TSAb], antithyroglobulin antibody [TgAB], and antithyroid peroxidase antibody [TPOAB]), and they still have regular outpatient follow‐ups after being discharged from the hospital.

In addition, the main examination items that needed to be completed included the routine sequence of orbital MRI, RESOLVE DWI, and SPECT/CT technetium (^99m^TcO4‐) imaging of the thyroid. All patients were instructed to abstain from smoking.

The orbital MRI scans were performed using a VIDA 3.0 T scanner and a 12‐channel phased‐array head coil (Siemens, Erlangen, Germany) and included the following sequences. T2‐weighted imaging fat suppression (T2WI‐FS) sequence: orbital transverse position (TR, 4000 ms; TE, 79 ms), coronal position (TR, 3800 ms; TE, 79 ms), oblique sagittal position (TR, 3500 ms; TE, 79 ms), T1‐weighted imaging (T1WI), and orbital transverse section (TR, 600 ms; TE, 10 ms) scan. The positioning line of the transverse scan is parallel to the auditory canthus line, the coronal scan is perpendicular to the hard palate, and the oblique sagittal scan is parallel to the long axis of the optic nerve. The slice thickness was 3 mm, the slice spacing was 0 mm, the FOV was 200 mm × 160 mm, and the scan matrix was 384 × 384. The orbital MRI conventional images mainly contain transverse section protrusion of the eyeball, lacrimal gland morphological parameters (transverse sectional protrusion [mm], lacrimal gland length [mm], width [mm] and area [mm^2^], and coronal section lacrimal gland length [mm], width [mm], and area [mm^2^]).

RESOLVE DWI parameters were as follows: TR = 1700 ms, TE = 102 ms, b‐value: 01,000 s/mm^2^, FOV: 250 mm × 250 mm, slice thickness 3 mm, slice spacing 0 mm, NEX 1, and scan time 2 min 20 s. RESOLVE DWI was used to measure the apparent diffusion coefficient (ADC) value (× 10^−3^ mm^2^/s) of the lacrimal gland.

Three physicians with over 10 years of experience in head and neck imaging diagnosis analyzed all the images using Siemens postprocessing workstations. The average values of each patient’s parameters for both eyes were taken as the observation value for analysis.

Because the orbital part of the lacrimal gland and the eyelid part are not easily distinguishable on images, they were measured together in the study [22]. The specific parameter measurement methods are as follows, as shown in Figure 1. The illustration shows the patients in the GO group before I‐131 treatment. ① On the transverse image of T2WI‐FS, the maximum layer of the eyeball and optic nerve was selected for display. The vertical distance from the corneal apex to the line connecting the leading edges of the zygomatic arches on both sides was measured as the proptosis (23.0 mm for the left eye and 23.4 mm for the right eye, Figure 1(a)). The vertical distance from the front ends of the lacrimal glands on both sides to the front ends of the zygomatic arches on both sides was measured as the lacrimal gland protrusion (13.7 mm for the left eye and 13.0 mm for the right eye, Figure 1(c)) [23]. ② Based on the transverse image of T2WI‐FS, the maximum level of the lacrimal gland display was selected. The distance from the leading edge (or the uppermost edge) to the last edge (the lowest edge) of the lacrimal gland was measured as the transverse/coronal long diameter of the lacrimal gland, and the distance perpendicular to the innermost and outer edges of the transverse/coronal long diameter was the short diameter of the lacrimal gland. We manually delineated the lacrimal gland boundary at this level to obtain the maximum cross‐sectional area in the transverse and coronal positions (transverse lacrimal gland: long diameter, short diameter, and area: 26.2 mm, 7.3 mm, and 166.80 mm^2^ for the left eye, and 27.7 mm, 7.1 mm, and 164.45 mm^2^ for the right eye, respectively; see Figure 1(e); coronal lacrimal gland: long diameter, short diameter, and area: left eye 18.2 mm, 7.5 mm, and 122.66 mm^2^, and right eye 20.5 mm, 7.5 mm, and 140.63 mm^2^, respectively; see Figure 1(g)). ③ Measurement of the ADC value of the lacrimal gland [24]. The obtained original images were input into the background processing workstation. By comparing T2WI‐FS and RESOLVE DWI images, the maximum lacrimal gland plane was selected on the ADC image, and a circular region of interest (ROI) (area 5–10 mm^2^) was manually delineated to obtain ADC values (left eye: 1.303 × 10^−3 ^mm^2^/s; right eye: 1.425 × 10^−3 ^mm^2^/s; Figure 1(k)).

FIGURE 1Comparison of orbital MRI images of the same patients with GO patient before and after iodine‐131 treatment. (a, c, e, g, i, k) Measurements before iodine‐131 treatment for a GO patient. (b, d, f, h, j, l) MRI images of the orbits of GO patients whose thyroid function returned to normal or decreased within one year after radioactive iodine‐131 treatment. (a‐b) Diagram showing the measurement of exophthalmos in the cross‐sectional T2WI‐FS images of GO patients before and after iodine‐131 treatment. Before I‐131 treatment, proptosis was 23.0 mm in the left eye and 23.4 mm in the right eye (Figure 1(a)). After I‐131 treatment, proptosis was 20.0 mm in the left eye and 20.0 mm in the right eye (Figure 1(b)). (c‐d) Diagram showing the measurement of lacrimal gland protrusion before and after iodine‐131 treatment in patients with GO (from transverse position in T2WI‐FS). The lacrimal gland prominence was 13.7 mm in the left eye and 13.0 mm in the right eye before treatment (Figure 1(c)), and 13.0 mm in the left eye and 12.4 mm in the right eye after treatment (Figure 1(d)). (e‐f) Diagram showing the measurement of the long diameter, short diameter and area of the lacrimal gland by cross‐sectional T2WI‐FS before and after iodine‐131 treatment in GO patients. On axial images, before treatment, the long and short diameters of the lacrimal glands were 26.2 and 7.3 mm in the left eye (area: 166.8 mm^2^), and 27.7 and 7.1 mm in the right eye (area: 164.45 mm^2^) (Figure 1(e)). After treatment, the long and short diameters of the lacrimal glands were 25.1 and 7.2 mm in the left eye (area: 152.73 mm^2^), and 25.8 and 6.8 mm in the right eye (area: 130.08 mm^2^) (Figure 1(f)). (g‐h) Transverse DWI images of the lacrimal gland of GO patients before and after iodine‐131 treatment. On T2WI‐FS coronal images, before treatment, the long and short diameters of the lacrimal glands were 18.2 and 7.5 mm in the left eye (area: 122.65 mm^2^), and 20.5 and 7.5 mm in the right eye (area: 140.63 mm^2^) (Figure 1(g)). After treatment, the long and short diameters of the lacrimal glands were 18.4 and 7.6 mm in the left eye (area: 112.89 mm^2^), and 18.7 and 6.5 mm in the right eye (area: 122.66 mm^2^) (Figure 1(h)). (k‐l) Schematic diagram showing the measurement of ADC values on the cross‐sectional ADC maps of GO patients before and after iodine‐131 treatment. Before treatment, the ADC value were 1303 ×10‐3 mm^2^/s in the left eye and 1435 ×10^−3^ mm^2^/s in the right eye (Figure 1(k)). After treatment, the ADC value were 1088 ×10^−3^ mm^2^/s in the left eye and 1178 ×10^−3^ mm^2^/s in the right eye (Figure 1(l)). Note: GO: Graves ophthalmopathy; T2WI‐FS: T2‐weighted imaging for fat inhibition; DWI: Diffusion weighted imaging; ADC: Apparent diffusion coefficient.

**Figure:**
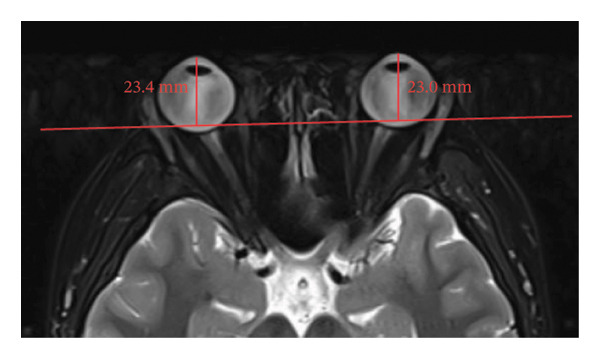
(a)

**Figure:**
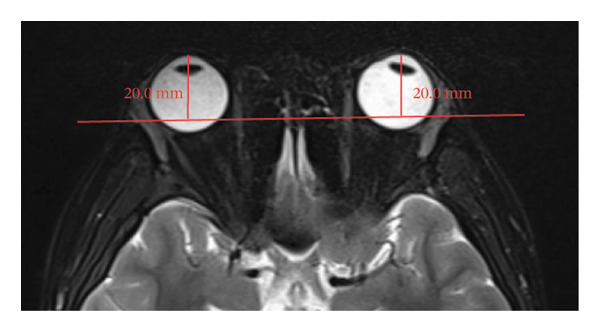
(b)

**Figure:**
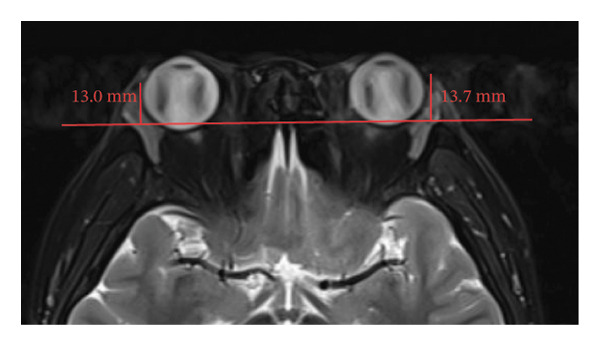
(c)

**Figure:**
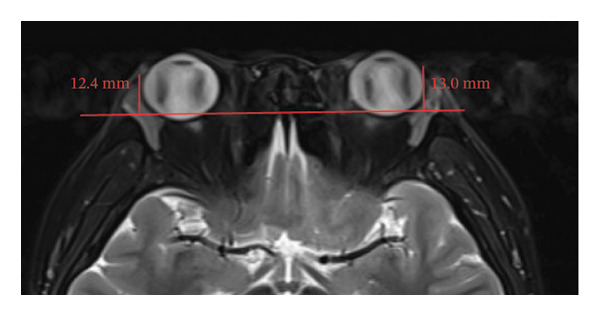
(d)

**Figure:**
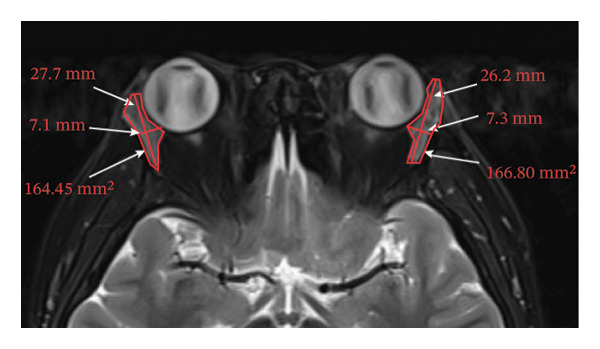
(e)

**Figure:**
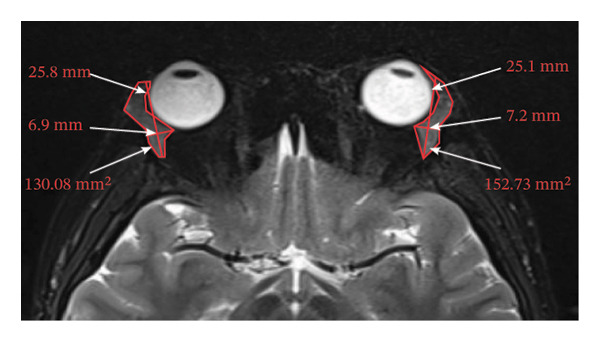
(f)

**Figure:**
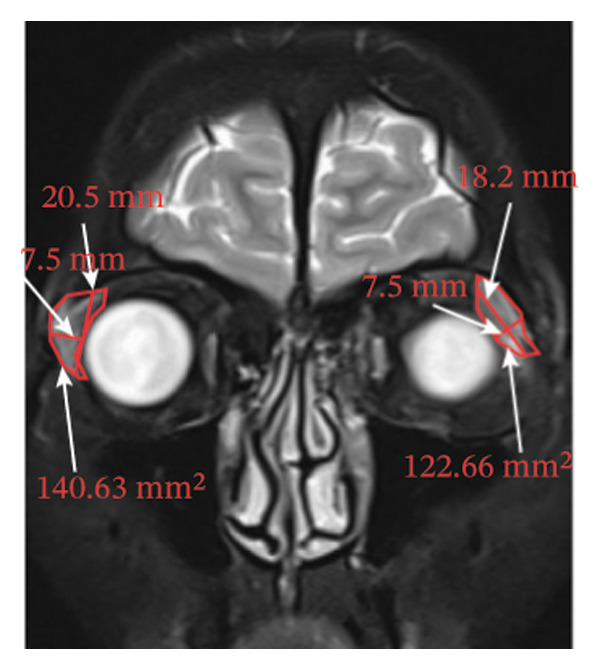
(g)

**Figure:**
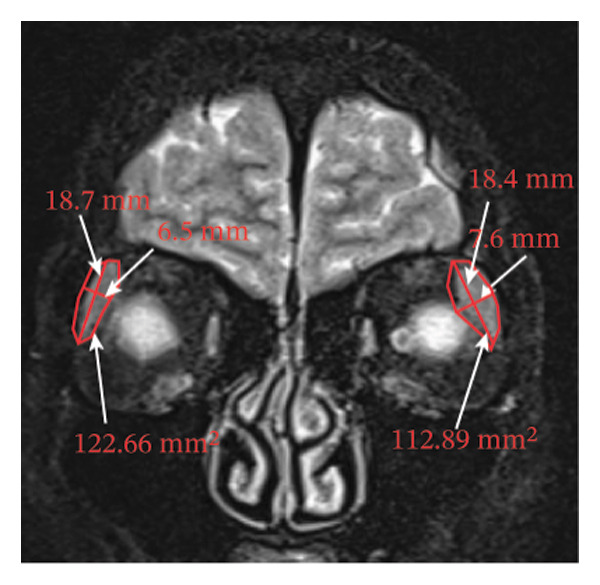
(h)

**Figure:**
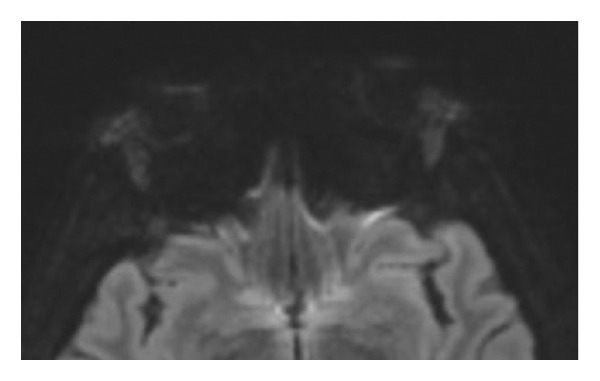
(i)

**Figure:**
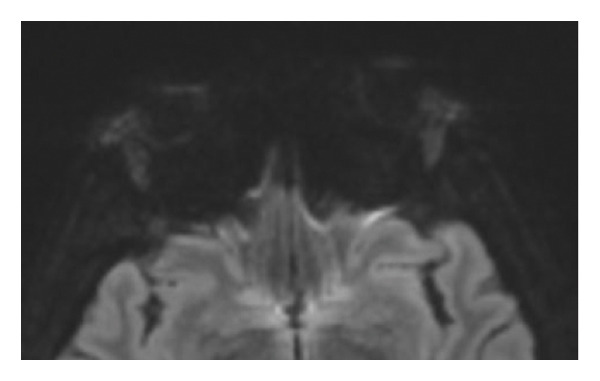
(j)

**Figure:**
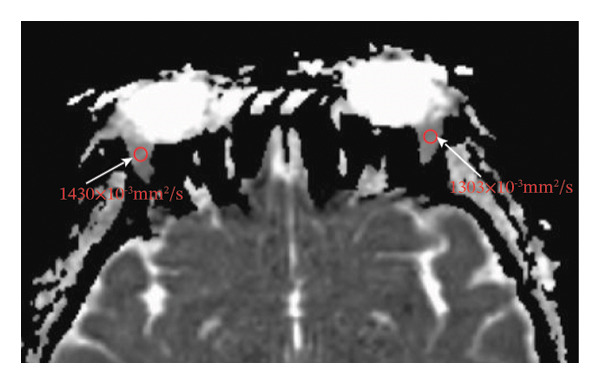
(k)

**Figure:**
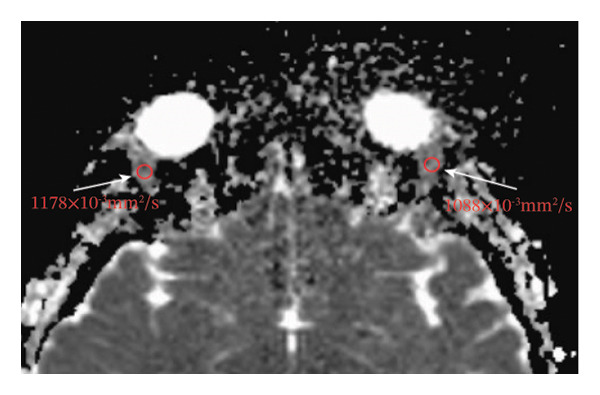
(l)

SPECT/CT was performed using an intravenous injection of 5.0 mCi of ^99m^TcO_4_^-^ and a Discovery NM670 dual‐head gamma camera (GE Healthcare, Waukesha, WI, USA) equipped with a low‐energy high‐resolution parallel‐hole collimator. The following settings were used: energy peak 140 keV, matrix size 256 × 256, window width 20%, magnification 2.57, and acquisition count 500 K.

**FIGURE 2:**
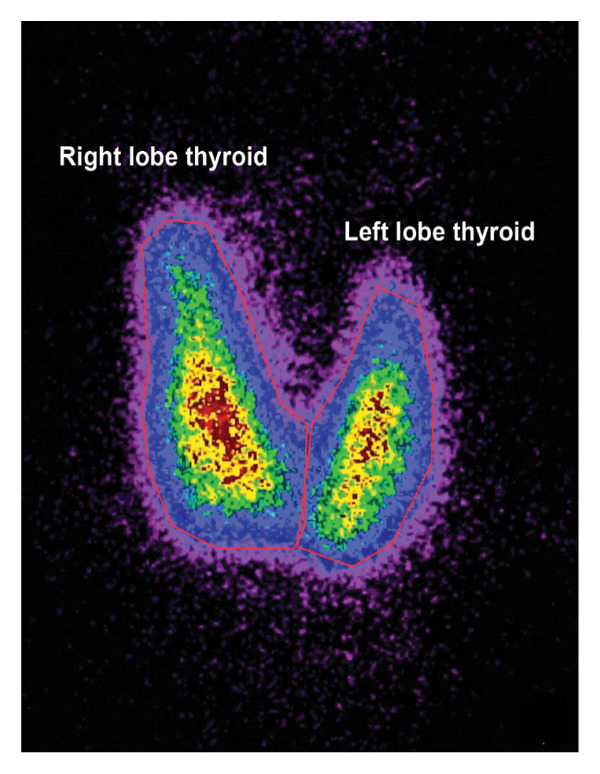
Schematic diagram of postprocessing for measuring thyroid weight and area by SPECT/CT and the thyroid technetium (^99m^TcO4‐) imaging.

For the calculation of thyroid ^99m^TcO4‐imaging weight, the postprocessing system configured in the SPECT instrument was used, and the edges of the left and right lobes of the thyroid were manually delineated (between red and blue) to obtain the thyroid weight (*g*) and the thyroid gland area (cm^2^). At the same time, the system automatically calculates the thyroid technetium uptake rate (%) (Figure 2).

All patients with GO and GD should not receive additional antithyroid drugs within 3 months after oral administration of radioactive I‐131 solution. Thyroid function should be reexamined multiple times within 1 month to 1 year. When thyroid function returns to cure or hypothyroidism after radioactive I‐131 treatment, orbital MRI examination and SPECT/CT thyroid ^99m^TcO4‐ imaging should be reexamined.

It can be seen that the average, median, and interquartile range of MR interval time in the GO and GD groups of patients are all very close. A two‐independent sample *t*‐test showed *t* = 0.740, *p* = 0.461  > 0.05; on orbital MR inspection, there was no statistically significant difference time interval.

### 2.3. Statistical Analysis

Data analysis was performed using SPSS 24.0 (IBM, Armonk, NY, USA). Continuous data conforming to the normal distribution (according to the Shapiro–Wilk test) were presented as means ± standard deviations. The male‐to‐female ratio in Table 1 was tested using the Pearson chi‐square test, and the other data were analyzed using the two independent samples *t*‐test. The data in Table 2 were analyzed using the paired sample *t*‐test. The Spearman correlation coefficient, *r*, was used to determine the strength of the correlation: *r* ≥ 0.8 indicated a very strong correlation, 0.8 > *r* ≥ 0.6 indicated a strong correlation, 0.6> *r* ≥ 0.4 indicated a moderate correlation, and *r* < 0.4 indicated a weak correlation [25]. Two‐sided *p* values < 0.05 were considered statistically significant.

**TABLE 1:** Comparison of general data, orbital MRI parameters, and thyroid SPECT/CT parameters before iodine‐131 treatment between GO group and GD group.

Variables	GO group (*n* = 41)	GD group (*n* = 32)	*χ* ^2^value/*t* value	*p* value
Age	40.29 ± 12.96	41.40 ± 11.64	−0.505	0.616
Male/female	19/22	14/18	0.049	0.825
Transverse eyeball protrusion, mm	18.24 ± 1.24	17.72 ± 2.22	1.188	0.241
Transverse lacrimal gland protrusion, mm	13.65 ± 1.47	12.90 ± 2.62	1.452	0.153
Transverse lacrimal gland ADC, mean value/(× 10^−3^ mm^2^/s)	1.458 ± 2.01	1.333 ± 1.98	2.657	0.010
MRI transverse lacrimal gland length, mm	17.21 ± 2.29	15.61 ± 2.54	2.851	0.006
MRI transverse lacrimal gland width, mm	5.25 ± 0.92	4.95 ± 1.03	1.303	0.197
MRI transverse lacrimal gland area, mm^2^	93.05 ± 15.66	87.86 ± 21.55	1.191	0.238
MRI coronal lacrimal gland length, mm	17.99 ± 2.71	17.84 ± 3.50	0.207	0.836
MRI coronal lacrimal gland width, mm	5.23 ± 0.89	5.02 ± 0.72	1.119	0.267
MRI coronal lacrimal gland area, mm^2^	109.81 ± 20.52	108.06 ± 25.47	0.324	0.747
SPECT/CT thyroid weight, g	53.53 ± 27.28	45.11 ± 19.75	1.471	0.146
SPECT/CT thyroid area, cm^2^	28.08 ± 10.25	24.40 ± 8.80	1.620	0.110
SPECT/CT^99m^Tc‐pertechnetate thyroid uptake (%)	17.82 ± 11.34	16.81 ± 6.76	0.476	0.636

*Note:* GO = Graves’ ophthalmopathy (or Graves’ eye disease).

Abbreviations: ADC = apparent diffusion coefficient, GD = Graves’ disease, MRI =  magnetic resonance imaging, SPECT/CT = single‐photon emission computed tomography/computed tomography.

**TABLE 2:** Comparison of orbital MRI parameters and thyroid SPECT/CT parameters of 41 GO patients before and after iodine‐131 treatment.

Variables	Before iodine‐131 treatment	After iodine‐131 treatment	*p*
Age	40.29 ± 12.96	—	—
Male/female	19/22	—	—
Transverse eyeball protrusion, mm	18.24 ± 1.24	18.19 ± 1.22	0.202
Transverse lacrimal gland protrusion, mm	13.65 ± 1.47	12.02 ± 1.25	≤ 0.001
Transverse lacrimal gland ADC, mean value/(× 10^−3^ mm^2^/s)	1.458 ± 2.01	1.342 ± 2.13	≤ 0.001
MRI transverse lacrimal gland length, mm	17.21 ± 2.29	16.03 ± 2.28	≤ 0.001
MRI transverse lacrimal gland width, mm	5.25 ± 0.92	5.23 ± 0.90	0.448
MRI transverse lacrimal gland area, mm^2^	93.05 ± 15.66	84.68 ± 14.76	≤ 0.001
MRI coronal lacrimal gland length, mm	17.99 ± 2.71	17.67 ± 2.61	≤ 0.001
MRI coronal lacrimal gland width, mm	5.23 ± 0.89	5.20 ± 0.91	0.129
MRI coronal lacrimal gland area, mm^2^	109.81 ± 20.52	104.50 ± 19.03	≤ 0.001
SPECT/CT thyroid weight, g	53.53 ± 27.28	48.22 ± 23.65	≤ 0.001
SPECT/CT thyroid area, cm^2^	28.08 ± 10.25	24.89 ± 8.74	≤ 0.001
SPECT/CT^99m^Tc‐pertechnetate thyroid uptake (%)	17.82 ± 11.34	15.14 ± 9.57	≤ 0.001

*Note:* GO = Graves’ ophthalmopathy (or Graves’ eye disease).

Abbreviations: ADC = apparent diffusion coefficient, MRI = magnetic resonance imaging, SPECT/CT = single‐photon emission computed tomography/computed tomography.

## 3. Results

### 3.1. Comparison of General Data, Orbital MRI Parameters, and Thyroid SPECT/CT Parameters Before I‐131 Treatment Between GO Group and GD Group

Before the radioactive I‐131 treatment, there were statistically significant differences in the cross‐sectional ADC values and the long diameter of the lacrimal glands between the GO and GD groups (*p* < 0.05). There were no statistically significant differences in gender, age, other MRI parameters, and SPECT/CT thyroid technetium uptake imaging parameters (*p* > 0.05) (Table 1).

### 3.2. Basic Characteristics and Orbital Quantitative Parameters Before and After I‐131 Treatment

This study included 41 GO patients (19 males, mean age 40.29 ± 12.96). The following parameters decreased after radioactive I‐131 treatment compared with before treatment: MRI transverse diameter of lacrimal gland protrusion (13.65 ± 1.47 vs. 12.02 ± 1.25, *p* < 0.001), mean ADC value of the lacrimal gland (1.458 ± 2.01 vs. 1.342 ± 2.13, *p* < 0.001), transverse lacrimal gland length (17.21 ± 2.29 vs. 16.03 ± 2.28, *p* < 0.001), transverse lacrimal gland area (93.05 ± 15.66 vs. 84.68 ± 14.76, *p* < 0.001), coronal lacrimal gland length (17.99 ± 2.71 vs. 17.67 ± 2.61, *p* < 0.001), and coronal lacrimal gland area (109.81 ± 20.52 vs. 104.50 ± 19.03, *p* < 0.001). In addition, thyroid weight (53.53 ± 27.28 vs. 48.22 ± 23.65, *p* < 0.001), thyroid area (28.08 ± 10.25 vs. 24.89 ± 8.74, *p* < 0.001), and thyroid technetium uptake (17.82 ± 11.34 vs. 15.14 ± 9.57, *p* < 0.001) measured by SPECT/CT were also decreased (Table 2).

**FIGURE 3:**
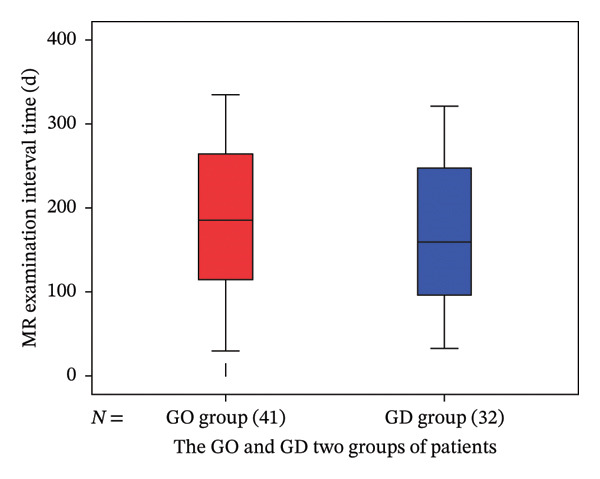
Box plots of the time intervals between orbital magnetic resonance examinations before the start of radioactive iodine‐131 treatment and at the time of cure or hypothyroidism in patients with GO and GD. Note: GO: Graves’ ophthalmopathy; GD: Graves’ disease.

The interval for follow‐up of orbital MRI is shown in Figure 3 and Table 3.

**TABLE 3:** The statistical data on the time intervals between orbital magnetic resonance examinations for patients with GO and GD.

	Minimum value (day)	Maximum value (day)	Lower quartile (day)	Middle quartile (day)	Upper quartile (day)
GO (*n* = 41)	30.0	335.0	104.5	186.00	268.00
GD (*n* = 32)	33.0	321.0	93.25	160.00	252.00

Abbreviations: GD = Graves’ disease, GO = Graves ophthalmopathy.

It can be seen that the average, median, and interquartile range of MR reexamination time in the GO and GD groups of patients are all very close, and *t* = 0.740, *p* = 0.461 > 0.05; on orbital MR inspection, there was no statistically significant difference time interval.

### 3.3. Correlations

The difference in thyroid weight before and after I‐131 treatment in patients with GO was positively correlated with the changes in lacrimal gland protrusion, transverse lacrimal gland length, transverse lacrimal gland area, coronal lacrimal gland length, coronal lacrimal gland area, and mean ADC value of the lacrimal gland. The correlation coefficients were *r* = 0.600, 0.748, 0.678, 0.683, 0.560, 0.766 (Figure 4).

FIGURE 4Correlation graph of changes in MRI parameters of the lacrimal gland and changes in thyroid weight before and after iodine‐131 treatment in 41 patients with Graves’ ophthalmopathy. (a) The correlation graph showing the difference in thyroid weight before and after ^131^I treatment in patients with GO and the change value of lacrimal gland protrusion. (b) The correlation graph showing the difference in thyroid weight before and after ^131^I treatment in patients with GO and the change value of the transverse lacrimal gland length. (c) The correlation graph showing the difference in thyroid weight before and after ^131^I treatment in patients with GO and the change value of the transverse lacrimal gland area. (d) The correlation graph showing the difference in thyroid weight before and after ^131^I treatment in patients with GO and the change value of the coronal lacrimal gland length. (e) The correlation graph showing the difference in thyroid weight before and after ^131^I treatment in patients with GO and the change value of the coronal lacrimal gland area. (f) The correlation graph showing the difference in thyroid weight before and after ^131^I treatment in patients with GO and the change value of the transverse lacrimal gland mean ADC value. Note: MRI: magnetic resonance imaging; GO: Grave’s ophthalmopathy.

**Figure:**
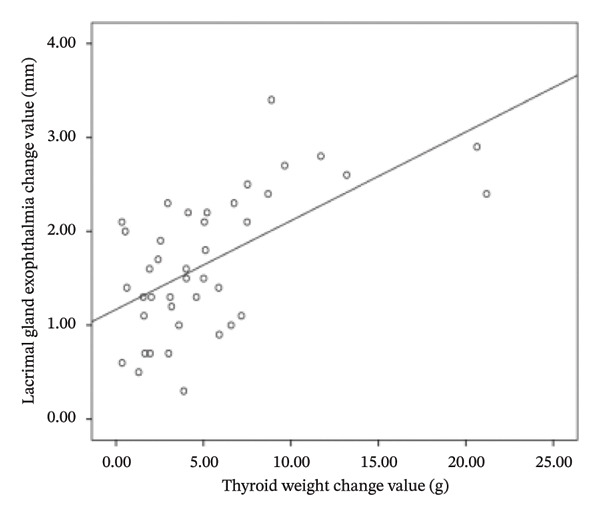
(a)

**Figure:**
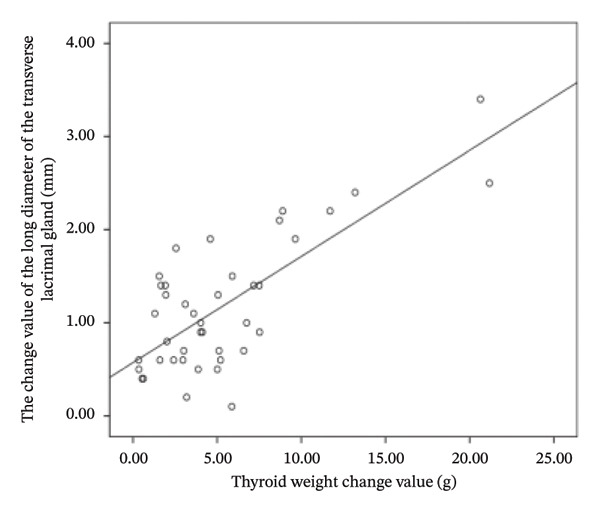
(b)

**Figure:**
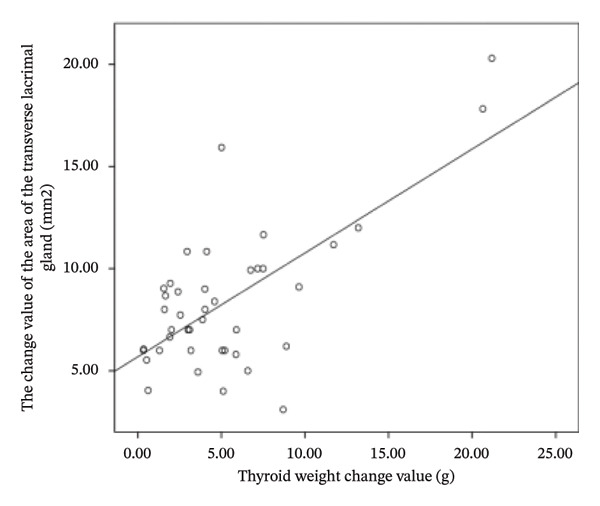
(c)

**Figure:**
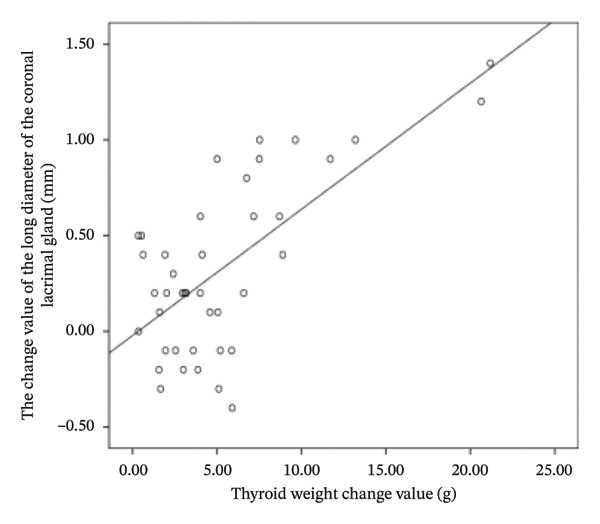
(d)

**Figure:**
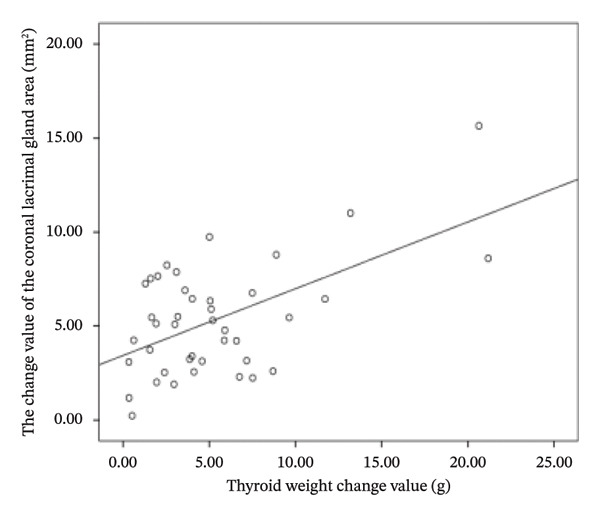
(e)

**Figure:**
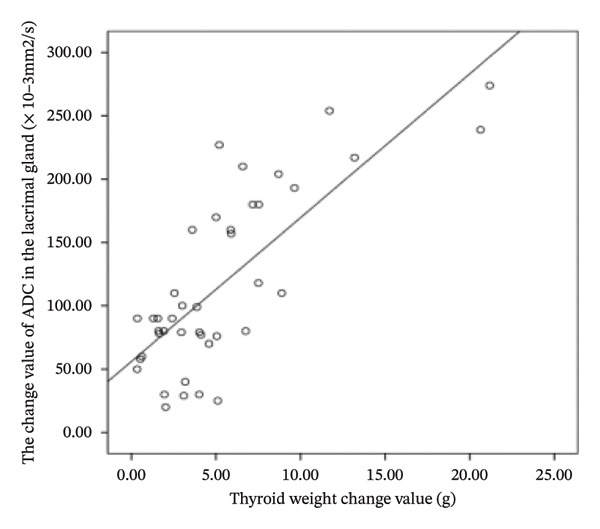
(f)

## 4. Discussion

The results suggested that in patients with GO, lacrimal gland protrusion, MRI lacrimal gland length and area, and mean lacrimal gland ADC value may be reliable imaging indicators for evaluating the prognosis of I‐131 treatment.

GO is a condition in patients with GD accompanied by inflammatory changes in the orbital soft tissues, affecting 25%–40% of patients with GD [8, 26]. The most common and prominent clinical manifestation of GO is proptosis, affecting 91.24% of patients, followed by eyelid retraction (83.33%), eyelid swelling (79.38%), and enlargement of the extraocular muscles (75.42%) [8]. Proptosis in GO is primarily caused by the expansion of orbital fat and/or extraocular muscles [15]. In this study, the average proptosis degree in the GO group was 18.17 ± 1.67 mm, which was lower than that reported by Bartalena et al. [8] and Inoue et al. [27]

There was no significant difference in the degree of eye protrusion between the GD and GO groups in this study, but the proptosis in patients with GO was slightly greater than that reported by Ji et al. [13] and Wang et al. [28] for inactive GO but slightly less than the proptosis observed in active GO. The differences may be related to the varying clinical activity and severity of GO across different studies. In this study, the patients with GO were in the inactive stage and had a mild clinical severity. The degree of proptosis was not obvious and was not much different from that of the GD patients in this study.

Cevik et al. [29] reported that the average left eye proptosis in their GO group was significantly higher than that in controls. However, considering that ocular involvement in GO patients predominantly affects both eyes, the study data were analyzed using the average values of both eyes as the analytical factor. Zhang et al. [30] treated 37 active GO patients with glucocorticoids, and the degree of proptosis before and after treatment was greater than the data reported in this study. The analysis suggests that the results may be related to the inactive phase of the GO patients enrolled in this study, but all conclusions indicate that the differences in proptosis between before and after treatment were not statistically significant.

However, Yang et al. [31] used three different medications (glucocorticoids, cyclophosphamide, and octreotide) to treat patients with GO. They found that the proptosis in the glucocorticoid and cyclophosphamide groups was lower than that in the octreotide group, and the changes in proptosis among the three treatment methods were all statistically significant. These results differ from the findings of this study, showing no statistically significant differences in proptosis before and after I‐131 treatment. The reasons could include the patient population and the nature of the treatments. Indeed, I‐131 treatment is primarily used to treat thyroid abnormalities, while glucocorticoid, cyclophosphamide, and octreotide are systemic treatments that can also affect the orbit.

Xiao et al. [32] suggested that proptosis did not affect the quality of life in patients with GO. On the other hand, Törring et al. [33] indicated that proptosis, along with intraocular pressure, vision, eyelid retraction, and other ocular signs, did have a significant impact on the quality of life in patients with GO. Proptosis is also considered a risk factor for ocular surface damage [34]. The proptosis degree in patients with GO is positively correlated with intraocular pressure [35]. Therefore, it is essential not to overlook the detection of proptosis to prevent ocular surface damage and high intraocular pressure in patients with GO. Accurate, simple, noninvasive, and repeatable MRI examinations can facilitate the early diagnosis of GO, identify high‐risk patients, and assess treatment efficacy. MRI could be recommended as a method for evaluating proptosis.

The prominence of the lacrimal gland is an imaging measurement that has received attention later than that of the extraocular muscles [22] and may be involved in and contribute to ocular surface damage in GO [36]. Numerous international authors have investigated this issue. Tan et al. [37] found that increased orbital fat in patients with GD could lead to lacrimal gland protrusion or prolapse. Inoue et al. [27] reported that the swelling rate of the lacrimal gland in patients with GO exceeded 70%. Harris et al. [38] found that lacrimal gland protrusion was mildly correlated with proptosis. Gagliardo et al. [22] reported that in patients with GO, lacrimal gland protrusion was significantly higher during the active phase compared with the inactive phase, and the protrusion in the inactive phase was still higher than that in controls. Gao et al. [16] reported that the degree of lacrimal gland protrusion was positively correlated with the CAS in patients with GO. In this study, during the 12‐month follow‐up after I‐131 treatment, the transverse diameter of lacrimal gland protrusion in patients with inactive GO was reduced compared with before treatment. Ji et al. [13] reported that patients with GO exhibited significant enlargement of the lacrimal gland, especially during the active phase of GO. The area and length of the lacrimal gland on both coronal and transverse views were higher compared with those of the control group. Chen et al. [39] believe that lacrimal gland protrusion could be an effective imaging marker for predicting the efficacy of hormone therapy in GO.

The application of MRI in orbital diseases is becoming increasingly widespread [40]. Kilicarslan et al. [41] used RESOLVE DWI to measure the extraocular muscles in patients with GO. Razek et al. [42] reported that the mean ADC values of the lacrimal gland could serve as a diagnostic indicator for GO and as an assessment metric for determining disease activity. Cao et al. [43] found that the ADC values of the lacrimal gland in patients with active GO were higher than those in inactive GO. Combining fractional anisotropy (FA) and ADC yielded the best performance and sensitivity for distinguishing disease activity, while using ADC alone provided the highest specificity. Compared with the performance, sensitivity, and specificity of measuring lacrimal gland structure on conventional MRI, as reported by Hu et al. [44], these metrics showed significant improvement. However, there are very few studies conducted by both domestic and foreign scholars that utilize the RESOLVE DWI technique to measure the lacrimal glands and then evaluate the therapeutic effect of I‐131 treatment in patients with GO. This study indicates that the protrusion degree of the lacrimal gland and the ADC value of the lacrimal gland can be regarded as one of the reliable indicators for diagnosing GO and can also be used as one of the evaluation indicators for the therapeutic effect of radioactive I‐131 treatment.

Deng et al. [18] reported that after I‐131 treatment, thyroid volume, technetium uptake, and thyroid function were reduced compared with the pretreatment levels. These parameters are valuable for assessing the effectiveness of I‐131 treatment in GD. Hu and Shi [45] confirmed that thyroid weight was a definitive factor influencing the efficacy of I‐131 treatment for GD. Kim et al. [46] showed that the technetium uptake rate and functional thyroid mass before treatment were independently associated with the efficacy of hyperthyroidism treatment in patients with GO. These previous studies support the results observed here, i.e., that SPECT/CT measurements of functional thyroid weight, area, and technetium uptake rate were significantly reduced after I‐131 treatment compared with pretreatment levels in patients with GO. These differences could serve as indicators for evaluating the efficacy of I‐131 treatment in patients with GO. In this study, there was no statistically significant difference in the SPECT/CT thyroid technetium (^99m^TcO4‐) imaging estimations of thyroid weight, area, and technetium uptake rate between the GD and GO groups. Analyzing the reasons, it might be related to the fact that the GO patients in this study were in the inactive period of CAS, resulting in little difference in thyroid imaging results between the GO patients and the GD patients.

### 4.1. Limitations

This study was not without limitations. First, there has been no study on the changes in thyroid function and autoantibodies in patients with GO after radioactive iodine treatment and their relationship with MR lacrimal gland parameters. Second, no studies have been conducted on the changes in other ocular structures such as extraocular muscles in patients with GO after radioactive I‐131 treatment and their correlation with the lacrimal gland. Third, this study could not combine ^99m^TcO4‐octreotide orbital imaging and MRI orbital examinations for the diagnosis and staging of GO. Fourth, there was a lack of further investigation into the relationship between MRI parameters in patients with GO and ocular surface conditions (such as meibomian gland dysfunction and dry eye syndrome). Fifth, the number of patients was limited, preventing the study from examining changes and the significance of MRI lacrimal gland parameters in patients with GO after treatments such as systemic steroid therapy, intraorbital steroid injections, or orbital radiotherapy. Sixth, the study was cross‐sectional, preventing the analysis of causality. Finally, the study was retrospective, limiting the data to those available in the patient charts.

## 5. Conclusion

This study suggests that lacrimal gland protrusion, diameter, length, area of the lacrimal gland, and lacrimal gland ADC values in orbital MRI could serve as reliable imaging indicators for evaluating I‐131 treatment in patients with GO. Additional studies are necessary for confirmation.

## Author Contributions

Xing‐Wang Wu designed the study protocol and made significant revisions to the manuscript. Man Wang drafted and wrote the manuscript and acquired and analyzed the data for this study. Zhong‐Yuan Shen acquired, analyzed, or interpreted the data for this study and made revisions to the MRI‐related content of the manuscript.

## Funding

This work was supported by the Anhui Medical University 2024 University Scientific Research Plan Compilation (Natural Science) Project (grant number 2024AH050736).

## Disclosure

All authors have approved the final version of the manuscript to be published and agree to be accountable for all aspects of the work, ensuring the accuracy and integrity of the research.

## Ethics Statement

The study was approved by the Ethics Committee of Fuyang Hospital Affiliated to Anhui Medical University (KY2024068). All participants were informed about the study protocol and provided written informed consent to participate in the study. The authors confirm that all methods were performed in accordance with the relevant guidelines. All procedures were performed in accordance with the ethical standards laid down in the 1964 Declaration of Helsinki and its later amendments.

## Conflicts of Interest

The authors declare no conflicts of interest.

## Data Availability

All data generated or analyzed during this study are included in this published article.
